# Sustainable Poultry Nutrition Using Citric Acid By-Products from Rice to Boost Growth and Carcass Yield in Thai KKU 1 Broiler Chickens

**DOI:** 10.3390/ani14233358

**Published:** 2024-11-21

**Authors:** Mutyarsih Oryza.S, Padsakorn Pootthachaya, Nisakon Pintaphrom, Sirisak Tanpong, Narirat Unnawong, Anusorn Cherdthong, Bundit Tengjaroenkul, Sawitree Wongtangtintharn

**Affiliations:** 1 Department of Animal Science, Faculty of Agriculture, Khon Kaen University, Khon Kaen 40002, Thailand; mutyarsih.or@kkumail.com (M.O.); padsakornp@kkumail.com (P.P.); nisakon_m@kkumail.com (N.P.); sirisakt@kkumail.com (S.T.); nariratunnawong@kkumail.com (N.U.); anusornc@kku.ac.th (A.C.); 2 Department of Veterinary Public Health, Faculty of Veterinary Medicine, Khon Kaen University, Khon Kaen 40002, Thailand; btengjar@kku.ac.th

**Keywords:** agro-waste, broiler diet, growth, meat yield, carcass

## Abstract

This research aimed to enhance the diet of Thai broiler chickens (KKU 1) by assessing the impact of CABR on growth performance, carcass yield, and meat composition. A total of 320 one-day-old mixed-sex (1:1) Thai KKU 1 broiler chicks were randomly assigned to five dietary treatments incorporating CABR at levels of 0%, 3%, 6%, 9%, and 12%. The chicks were fed for a period of 56 days, divided into three phases: 1–21, 22–49, and 50–56 days. In the grower phase, birds receiving 3% and 6% CABR displayed the lowest feed conversion ratio (FCR) compared to other groups, while their body weight (BW) and BW gain (BWG) were significantly higher than those of other treatments (*p* < 0.05). In the finisher phase, performance differences were minimal, except for birds on the 3% CABR diet, which had greater BW than those on 9% or 12% CABR diets. Overall, throughout all phases, the inclusion of 3% CABR resulted in improved BWG and FCR compared to other groups across the study period (*p* < 0.05). Additionally, the 12% CABR treatment reduced dressing percentage compared to other groups, but it did not negatively impact the relative organ weights of the carcass or the quality of breast meat. Incorporating 3% and 12% CABR resulted in the highest values recorded for gross energy and ether extract in breast meat (*p* < 0.05). Furthermore, the gross energy of thigh meat increased with 3% to 12% CABR inclusion, peaking at the 6% CABR treatment (*p* < 0.05). This study found that adding 3% or 6% CABR to the diet of Thai broiler chickens improves their growth without negatively impacting meat or carcass quality.

## Simple Summary

Due to rising feed ingredient costs and the shift toward more sustainable agricultural practices, alternative feed sources and by-products are becoming increasingly important. Citric acid by-products from rice (CABR) show potential as a nutritious feed option that supports chicken production. This study compared the growth performance, carcass yield, and meat quality of broilers fed CABR-based diets with those on traditional feeds. The findings indicate that incorporating 3–6% CABR into broiler diets can deliver comparable results to conventional diets without compromising carcass quality. By adopting CABR, poultry farmers can reduce their reliance on standard feed ingredients, support a more sustainable food system, and better manage feed price volatility.

## 1. Introduction

In the absence of adequate management, agricultural residues—typically seen as little more than waste—can lead to pollution and other ecological issues. The citric acid manufacturing process results in the generation of citric acid waste, which amounts to millions of tons per year [1]. In the fermentation process, manufacturers create organic acids by using a variety of materials as substrates, such as cassava, maize, and rice starch [2]. Worldwide, citric acid production uses up to 60% of the substrate, according to the research [3]. One potential source for citric acid is plants; presently, the most common feedstocks for this process are corn and cassava. The production of citric acid in Thailand is often done using rice [1,2]. Rice is the predominant cereal and primary staple for the majority of the global population, particularly in Asian nations. Over the past decade, global rice production has grown at an average annual rate of 2.5 percent, resulting in a total production of 744.4 million tons in 2014. Rice is the primary locally produced crop in Thailand, with a total of 20.7 million metric tons in 2019 [4]. Therefore, rice has the potential to serve as an ingredient in the production of citric acid. Kudzai et al. [5] conducted an analysis of the capacity of rice and potato extracts to produce citric acid in order to satisfy the prevalent demand for this substance. The data demonstrated that rice was capable of producing the highest quantity of citric acid, and rice extract media was more beneficial than potato extract media. This underutilized resource holds immense potential because citric acid by-products still contain preserved organic acids, which could benefit gastrointestinal health and modulate immune responses in poultry [6]. Moreover, citric acid waste contains nutrients that are praised for their beneficial effects on poultry production. When used correctly with the right nutrition, management, and biosecurity measures, organic acids can be a very useful tool for keeping chickens’ digestive tracts healthy, which will improve their performance. Diets have incorporated citric acid due to its beneficial effects on the health and growth of broiler chickens [7]. Citric acid exhibits significant antimicrobial activity, enabling the preservation of feed against bacterial spoilage while simultaneously reducing the levels of undesirable bacteria in the gastrointestinal tract, ultimately improving growth rates [8,9]. Tanpong et al. [10] reported from a nutritional perspective that the by-product of citric acid from cassava contains 3588 kcal/kg of energy, 6.11% crude protein, and 0.71% citric acid content, all suitable for use in animal feed. Oryza et al. [4] describe the proximate composition and caloric content of citric acid by-products from rice (CABR) used for animal trials, stating that they contain 4005.72 kcal/kg of energy, 19.80% protein, and 3.30% citric acid content, making them suitable for potential animal feed use.

The Thai KKU 1 broiler chickens, a broiler crossbreed developed by the Network Center for Animal Breeding and Omics Research at Khon Kaen University, contain 25% Thai native chicken genetics [11,12]. This breed was designed to combine the resilience and adaptability of Thai native chickens with the fast growth rates of commercial broilers [13]. Thai native chickens, such as Pradu Hang Dam, are known for their unique flavor, firm texture, larger muscle fiber diameter, higher shear values, and lower fat content compared to broilers, though they are slower growing [12,13]. However, Thai KKU 1 broiler chickens successfully address the slow growth limitation of native chickens by incorporating commercial broiler traits [14]. The Thai KKU 1 broiler chicken aims to deliver high meat quality while reducing production costs, offering lower purine content compared to commercial broilers, which produce soft, tender meat [15]. This breed provides an optimal balance between productivity and meat quality, catering to modern consumer preferences for flavorful yet cost-effective poultry [16,17,18].

This indigenous chicken meat exhibits a unique taste and texture, commanding a higher market price than commercially available broilers in Thailand. The slower growth rate of indigenous chickens compared to their commercial counterparts could explain this difference in organoleptic properties [18]. By-products have advantages that are directly related to their low price as a feed, potentially reducing animal feed expenses when substituted. Despite these promising observations, developing a suitable diet specifically formulated to feed Thai broiler chickens with CABR remains critical due to the lack of existing information and research on this breed. Therefore, this study aims to improve the diet of Thai KKU 1 broiler chickens by evaluating the effects of CABR on growth performance, carcass yield, and meat composition.

## 2. Materials and Methods

### 2.1. Animal Ethics

All experimental protocols and procedures used in this research were reviewed and approved by the Institutional Animal Care and Use Committee of Khon Kaen University (record no. IACUC-KKU-69/63), based on the ethics of animal experimentation set forth by the National Research Council of Thailand.

### 2.2. Source of Citric Acid By-Product from Rice (CABR)

The liquid CABR was sourced from a factory in Eastern Thailand and sponsored by PS Nutrition Company Limited, based in Bangkok, Thailand. The by-products from the CABR, broken rice (BR), and rice bran (RB) were used as samples. The total weight of each sample was 50 kg, which was collected by random sampling using a tapered bag trier. The nutritional composition of CABR is provided in Table 1.

### 2.3. Animals and Experimental Design

This trial used 320 Thai KKU 1 broiler chickens of mixed sex (male-to-female ratio 1:1). The chickens were sourced from the Network Center for Animal Breeding and OMICS Research at the Faculty of Agriculture, Khon Kaen University, Thailand. The chickens were assigned to five different levels of citric acid waste product from rice: (1) the control group (0% inclusion of CABR), 3, 6, 9, and 12% CABR. The experimental diets were analyzed for chemical composition according to methods [19]. Table 1 presents the diets, which were calculated using a software program to adjust for balanced CP and metabolizable energy to meet the nutritional requirements of broiler or native chickens, as recommended by NRC [19], for the starter (1–21 days), grower (22–49 days), and finisher (50–56 days) phases.

### 2.4. Data Collection

#### 2.4.1. Performance Parameters

Body weight (BW) and feed intake (FI) were recorded. In each of the treatment groups, the BW of each bird was determined on a weekly basis using an electronic digital weighing machine. Then, the BW gain (BWG), feed conversion ratio (FCR), survival rate (SR) were also recorded. All birds from each treatment were weighed weekly using an electronic digital weighing machine to obtain the body weight. The amount of added feed to each pen and feed residue was recorded daily using the electronic digital weighing machine. Feed consumption was calculated on a per-period basis: (1) starter period (1–21 days), (2) growth period (22–49 days), (3) finishing period (50–56 days), and (4) overall period (1–56 days). BWG, FI, and FCR for each period were calculated. In all trials, mortality was recorded and reported as a cumulative percentage, while productive growth performance and survival rates were calculated following this formulation.

Productive growth performance:

$$\mathrm{BWG}=\frac{\mathrm{Final}\;\mathrm{weigh}\;\times \;\mathrm{initial}\;\mathrm{weight}\;}{\mathrm{number}\;\mathrm{of}\;\mathrm{birds}}$$

$$\mathrm{FI}=\frac{\mathrm{Total}\;\mathrm{feed}\;\mathrm{consumption}\;}{\mathrm{number}\;\mathrm{of}\;\mathrm{birds}\;}$$

$$\mathrm{FCR}=\frac{\mathrm{Feed}\;\mathrm{intake}\;}{\mathrm{Body}\;\mathrm{weight}\;\mathrm{gain}\;}$$

$$\mathrm{Survival}\;\mathrm{rates}\;(\%)=\frac{\mathrm{Number}\;\mathrm{of}\;\mathrm{initial}\;\mathrm{birds}-\mathrm{Number}\;\mathrm{of}\;\mathrm{dead}\;\mathrm{birds}\;}{\mathrm{Body}\;\mathrm{number}\;\mathrm{of}\;\mathrm{initial}\;\mathrm{birds}}$$

#### 2.4.2. Carcass Yield

The birds were fasted for 12 h prior to slaughter. At the end of the 56-day experiment, the birds were slaughtered to analyze the carcass quality. Two chickens from each pen with similar BW were selected and slaughtered to examine the carcass characteristics [20], minimizing variability related to size. The birds were then plucked to determine carcass yield, cuts (breast, sasami, drumstick, thigh, breast fillet), and edible viscera (heart, gizzard, and liver) according to the following formula:

$$\mathrm{CY}\;(\%)=\frac{\mathrm{Carcass}\;\mathrm{weight}\;\times 100}{\mathrm{Live}\;\mathrm{weight}}$$

#### 2.4.3. Meat Characteristic

Breast and thigh meat from each treatment were used for color measurement. The meat was filleted for three replications per treatment and analyzed under the International Commission on Illumination (CIE) Lab color space (CIELAB) system using a Chroma Meter CR-410 (Konica Minolta Sensing Inc., Tokyo, Japan).

Drip loss content was measured following the methods of Guo et al. [21] and Malila et al. [22]. Breast and thigh meat samples were filled 24 h postmortem, weighed, and stored in polyethylene trays covered with plastic film at 4 °C for 24 h. After that, exudate was discarded, the samples were weighed, and drip loss was calculated as initial weight (W1) minus final weight (W2), and expressed as a percentage:

$$\mathrm{Drip}\;\mathrm{loss}\;(\%)=\frac{\left[W1-W2\;\right]\times 100}{W1}$$

Following the method of Küçüközet and Uslu [23], cooking loss was determined in breast and thigh meat placed inside zip-top plastic bags in a water bath at 85 °C. The sample was cooked until the temperature of the meat reached 80 °C and then cooled down; the sample was weighed before and after the cooking process; and cooking loss was determined as the percentage of weight lost by the sample.

Cooking loss % = (Weight loss/Original meat weight) × 100

Breast and thigh meat from each treatment were cooked using a water bath (85 °C) until the meat temperature reached 80 °C; after the cooking process, the meat was cut to a diameter of 1 × 1 cm for each treatment for sensory evaluation using stable microsystem machines in England [24].

The chemical composition of carcass meat was determined using the AOAC method (1990). After a three-day drying procedure at 60 °C, the meat was ground and evaluated for crude protein, moisture, fat, and gross energy.

### 2.5. Statistical Analysis

The data were analyzed using a one-way analysis of variance (ANOVA) employing the general linear model (GLM) procedure in SAS [25]. A completely randomized design was used for all parameters. Prior to analysis, assumptions of normality and homogeneity of variance were checked. Normality was verified using the Shapiro-Wilk test, and homogeneity of variance was assessed using Levene’s test. Statistically significant differences among means were determined by Duncan’s new multiple-range tests, with significance accepted at *p* < 0.05. Outliers were detected by examining residuals and handled by removal if they significantly distorted the results. Missing or incomplete data were managed using listwise deletion, ensuring that only complete datasets were analyzed. Where appropriate, orthogonal polynomial contrasts were employed to evaluate the linear and quadratic trends associated with the increasing levels of dietary CABR. These contrasts were used to estimate the nature of the relationship (linear or curvilinear) between CABR levels and the key performance parameters, such as body weight (BW), body weight gain (BWG), feed intake (FI), and feed conversion ratio (FCR). A significance level of *p* < 0.05 was applied to test the significance of these trends. Data are expressed as means for each diet.

Additionally, the growth rate or body weight (BW) and feed conversion ratio (FCR) were visualized using R Studio version 10, which provided clear graphical representations of the data trends. The use of R Studio allowed for enhanced flexibility in visual analysis, contributing to a more comprehensive understanding of the statistical results.

## 3. Results

### 3.1. Growth Performance

**Figure 1:**
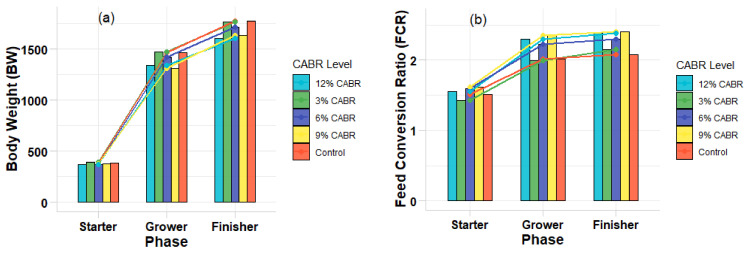
Effects of different dietary inclusions of a citric acid by-product from rice (CABR) on body (**a**) weight (BW) and (**b**) feed conversion ratio (FCR).

The growth performance of Thai KKU 1 broiler chicken on this diet is shown in Table 2 and Figure 1. The inclusion of CABR did not affect the performance of the birds during the starter phase (*p* > 0.05). However, in the grower phase, the FCR was lowest in birds fed 3% and 6% CABR compared to other treatments, while BW and BWG improved significantly compared to other groups (*p* < 0.05). During the finisher phase, we observed no significant changes in performance, with the exception of birds fed 3% CABR showing higher BW compared to other groups. Overall, across all phases, the inclusion of 3% CABR in the diet resulted in better BWG and FCR than the 9% or 12% CABR treatments throughout the entire experimental period (*p* < 0.05). Polynomial analysis further highlighted linear trends in BWG and FCR (*p* < 0.05) during the starter phase, suggesting a dose-dependent effect. In the grower phase, both linear (*p* = 0.002) and quadratic (*p* = 0.003) effects were observed for BW and BWG, with optimal performance at mid-level CABR inclusion (3–6%). Similarly, during the finisher phase, a significant linear trend was observed for BW (*p* < 0.05). During the whole time period, polynomial analysis confirmed significant linear effects in both BWG (*p* = 0.001) and FCR (*p* = 0.002). This showed that lower CABR levels (3%) were better than higher levels. In addition, feed cost value decreased by 2–5% when compared to the control group.

### 3.2. Carcass Yield

The utilization of CABR in carcass and internal organs in Thai KKU 1 broiler chickens is shown in Table 3. Carcass percentage was significantly affected (*p* < 0.05) when fed the 12% CABR treatment, which reduced the dressing percentage compared to other groups, but it did not negatively impact the relative organ weights of the carcass or the quality of breast meat (*p* > 0.05). The utilization of CABR as an energy source in the animal diet did not show any negative (*p* > 0.05) effect on carcass quality and relative organ weight.

### 3.3. Meat Quality

The effects of utilizing CABR on meat characteristics are presented in Table 4. The results indicated no significant effects (*p* > 0.05) on the L* (lightness) or a* (redness) values of Thai broiler meat. However, CABR had a significant effect (*p* < 0.05) on the b* (yellowness), indicating increased yellowness in the meat. As shown in Table 5, the chemical composition of the meat was also affected. CABR significantly increased (*p* < 0.05) the dry matter content of both breast and thigh meat, as well as the crude protein content in Thai broiler meat.

Polynomial analysis revealed significant linear trends for most parameters in meat composition. For breast meat, significant linear trends (*p* = 0.038) were observed for dry matter content, and gross energy followed a similar trend (*p* = 0.040). Ether extract levels also exhibited a significant linear increase (*p* = 0.035) with CABR inclusion. Quadratic trends were not significant for these parameters (*p* > 0.05).

In thigh meat, linear trends (*p* = 0.045) were observed for dry matter content, and gross energy exhibited a significant linear relationship (*p* = 0.003). Crude protein content in thigh meat also showed a linear trend (*p* = 0.021). Similar to breast meat, no significant quadratic effects were observed for thigh meat parameters (*p* > 0.05).

## 4. Discussion

The growth metrics (BW, BWG, FI, FCR, and SR) did not vary throughout the first phase of the study. Similarly, Oryza et al. [4] examined the by-products of CABR in broiler diets and discovered that substituting 3–6% of CABR for cornmeal had no impact on growth performance. During the phase 2 period and overall, the inclusion of citric acid by-products led to increased BW, BWG, and decreased FCR. This result is similar to the previous study by Hassan et al. [26], who reported that supplementation with citric acid in broiler diets led to enhanced BWG and lowered FCR. Similarly, Tanpong et al. [10] found similar results when they fed Japanese quails a by-product of CABR from cassava at levels of 3–12% for 1–6 weeks, reporting an increase in BWG and FI without other effects.

The BW was increased in comparison to the control groups. The improved BWG is probably due to the beneficial effects of organic acids on the gut flora, including improved digestion, absorption, and mucosal immunity [1,4]. The organic acids may affect the integrity of the microbial cell membrane or cell macromolecules or interfere with nutrient transport and energy metabolism, resulting in a bactericidal effect [10,27]. Moreover, due to its positive effects on feed intake (FI) and nutrient digestion, enhancing dietary fiber intake, it was also found that including organic acids in chicken rations increased performance. Specifically, this includes improvements in feed consumption and strengthened immune systems in the birds, potentially lowering disease risk [4,28]. Therefore, adding agricultural by-products from CABR to diets at levels of 3 to 6% could be an effective addition to animal feed. Perhaps the advantage of using the by-product as a feed additive could lead to a reduction in the overall cost of poultry feed, thereby reducing waste and promoting environmental sustainability.

Additionally, increasing the residual citric acid level in the diet to 12% resulted in a reduction of BW, BWG, and FI compared to the control groups. Most likely, the diet’s high crude fiber content caused the birds to increase their FI to compensate for their energy deficit. FI means, on the other hand, decreased at 3–6% of CABR over the overall period, but there was no impact; it might be that the palatability of the feed was reduced due to the high amount of crude fiber, and FI was reduced. A similar result was reported by Mehdikhany et al. [5], where means of FI decreased at the level of 2.5–5.0% of residual citric acid. The highest mean of SR in this study was at the level of 12% of CABR. According to Yadav et al. [29], using organic acids in the chicken diet enhanced productive performance, such as feed consumption, by improving the immune system of the birds, which could lower the risk of disease. This organic acid has the ability to reduce the risk of illness in feed, lower the pH of the intestinal tract, and eliminate harmful microorganisms by killing pathogenic bacteria [10]. Finally, organic acids, which can minimize dangerous microorganisms, improve poultry function by improving digestion and feed absorption. The FCR was significantly affected by the inclusion of CABR in this study. High-fiber diets typically result in relatively low energy density, which can potentially impact the feed conversion ratio of poultry [26]. Furthermore, this study did not significantly affect the productive index.

The quantity of CABR steadily increased, which resulted in the meat becoming more yellow in color. There was a correlation between the rise in lightness and the increase in the yellowness rating. This result aligns with the findings of Yadav et al. [29], who observed a comparable shift in yellowness (b*) values when fed with dried apple pomace and dried tomato pomace. Behera et al. [28] also reported the changed values of broiler meat yellowness when fed citrus waste. The increase in the yellowness value also suggests that the processing of citric acid in the raw materials mpacted the meat’s color. The chemical composition of feedstuffs used in animal diets also affects the color of the meat; the low pH of CABR’s chemical composition might be effective and increase the yellowness (b*) of the meat; likewise, Kralik et al. [30] reported that “light”color on breast meat (b* and a*) was considerable with lower pH. Similarly, the results from Dirinck et al. [31], who reported the use of citric acid waste in a diet, demonstrated that it had no significant effect on cooking loss, shear force, or drip loss compared to the control group, which did not use citric acid waste. There was no influence among the treatments in the diet, such as cooking loss and shear force of broiler meat, according to the findings of Angalet et al. [32], who assessed citrus sludge as a chicken diet and its effect on meat quality; the absence of observable differences between the treatments demonstrated this.

The 12% CABR treatment had a negative impact on the dressing percentage, which in turn led to a decrease in the birds’ BW, BWG, and FI. Additionally, the high crude fiber content of the diet may have caused the birds to increase their FI to offset their energy deficit, as fiber has been known to depress intake, increase bulkiness, and consequently cause growth depression and a negative impact on dressing percentage [28]. Compared to the control group, Tanpong et al. [10] found that citric acid by-products decreased BW and FI by 5.16 and 9.16% (BW) and 4.63 and 5.21% (FI) when they were at high levels of 9–12%. The quail fed throughout the 1–6 weeks showed decreases in BW, BWG, and FI when the addition of CABP to the diets reached the 9 and 12% ranges. These results confirm that CABR has a high fiber content, which could probably decrease birds’ carcass yield. From a commercial standpoint, the utilization of rich CABR feedstuffs in poultry diets may face certain limitations due to the fiber source and amount, as carcass characteristics determine the producer’s remuneration [33]. Shahin and El Azeem [34] indicated that birds fed a high-fiber diet had lower carcass weight than birds fed low-fiber diets. Despite the fact that adding citric acid by-products at levels of 0, 20, and 30% to swine diets improved carcass quality, the results did not significantly differ from the control group [35,36]. Similarly, using CABR as an energy source in animal diets did not negatively impact carcass yield or relative organ weights [37]. The study’s results showed that feeding 3 to 6% CABP as a replacement did not negatively affect carcass quality or organ weight, but it did enhance the dressing percentage and had no effect on the relative organ weight. At levels 3–6% in the diet, CABP could serve as an effective replacement. Similarly, CABR levels of 3–6% did not have any impact on its overall performance [1,4,10].

An increase in the percentage of moisture and a decrease in the percentage of ether extract in meat are factors that are correlated with an increase in crude protein [38]. The results that Mohammed et al. [39] provided on the impact of the utilization of organic acid on broilers indicated that it had a substantial influence on the percentage of moisture that was present in the meat. Crude protein increased relative to the control, whereas ether extract decreased in the meat. Reda [40] reported that the inclusion of fumaric acid in feed resulted in increased moisture and protein levels in broiler breast meat while simultaneously reducing ether extract concentration.

## 5. Conclusions

In conclusion, CABR can be used as an energy source in Thai KKU 1 broilers. The study shows that 3–6% CABR in the diet optimizes growth performance and FCR, while a higher inclusion of 12% CABR is detrimental. Therefore, it is recommended to limit CABR inclusion to 3–6% for optimal growth and meat quality. Further research should explore the long-term effects of CABR on carcass and meat quality, gut microbiota, and immune responses to improve its use in poultry nutrition. 

## Figures and Tables

**Table 1:** Feed ingredients of experimental diet during starter, grower, and finisher periods (as-fed basis).

Ingredients (%)	Starter (1–21 Days)					Grower (22–49 Days)					Finisher (50–56 Days)				
	Control	CABR				Control	CABR				Control	CABR			
		3%	6%	9%	12%		3%	6%	9%	12%		3%	6%	9%	12%
Corn meal	50.0	48.0	46.0	44.0	42.0	55.6	53.47	51.4	49.3	47.2	59.5	57.4	55.27	53.2	51.1
Soybean meal, 44% crude protein	26.9	25.8	24.7	23.6	22.5	19.3	18.3	17.3	16.3	15.3	12.5	11.5	10.5	9.5	8.5
Full-fat soybean	17.0	17.0	17.0	17.0	17.0	19.0	19.0	19.0	19.0	19.0	22.0	22.0	22.0	22.0	22.0
Dicalcium phosphate (P21%)	1.80	1.80	1.80	1.80	1.80	1.60	1.60	1.60	1.60	1.60	1.50	1.50	1.50	1.50	1.50
Limestone	1.60	1.60	1.60	1.60	1.60	1.40	1.40	1.40	1.40	1.40	1.30	1.30	1.30	1.30	1.30
DL-Methionine	0.250	0.250	0.250	0.250	0.250	0.200	0.200	0.200	0.200	0.200	0.170	0.170	0.170	0.170	0.170
L-Lysine	0.200	0.200	0.200	0.200	0.200	0.180	0.180	0.180	0.180	0.180	0.150	0.150	0.150	0.150	0.150
Rice bran oil	1.50	1.60	1.70	1.80	1.90	2.00	2.10	2.20	2.30	2.40	2.20	2.30	2.40	2.50	2.60
Salt	0.300	0.300	0.300	0.300	0.300	0.300	0.300	0.300	0.300	0.300	0.300	0.300	0.300	0.300	0.300
Choline chloride (60%)	0.100	0.100	0.100	0.100	0.100	0.100	0.100	0.100	0.100	0.100	0.100	0.100	0.100	0.100	0.100
Premix ^a^	0.350	0.350	0.350	0.350	0.350	0.350	0.350	0.350	0.350	0.350	0.350	0.350	0.350	0.350	0.350
Citric acid by-product from rice (CABR) ^b^	0.00	3.00	6.00	9.00	12.00	0.00	3.00	6.00	9.00	12.00	0.00	3.00	6.00	9.00	12.00
Total	100	100	100	100	100	100	100	100	100	100	100	100	100	100	100
Nutritional composition															
Crude protein, %	22.4	22.	22.3	22.2	22.1	20.1	20.0	20.0	20.0	19.9	18.3	18.3	18.2	18.2	18.17
Crude fiber	4.40	4.62	4.84	5.06	5.28	4.16	4.38	4.61	4.83	5.05	3.98	4.42	4.42	4.65	4.87
Calcium	1.67	1.44	1.34	1.53	1.21	5.56	5.83	5.51	5.83	5.51	6.17	7.76	7.85	7.92	7.88
Phosphorus	0.697	0.704	0.501	0.541	0.568	0.090	0.086	0.068	0.065	0.071	0.718	0.507	0.419	0.487	0.463
ME, kcal/kg	3013	3004	2994	2985	2975	3129	3118	3108	3097	3087	3209	3198	3188	3177	3167

^a^ Provided per kilogram of diet: 4.80 MIU of vitamin A, 2.00 MIU of vitamin D3, 30,000 IU of vitamin E, 1.20 g of vitamin K3, 1.20 g of vitamin B1, 3.20 g of vitamin B2, 2.00 g of vitamin B6, 0.0064 g of vitamin B12, 24.00 g of niacin, 0.80 g of folic acid, 0.08 g of biotin, and 6.00 g of pantothenic acid. 40.00 g of Zn, 48.00 g of Mn, 16.00 g of Fe, 6.40 g of Cu, 0.50 g of I, 0.04 g of Co, and 0.12 g of Se. 0.20 g of antioxidant, 0.88 g of anticaking agent, and 1.00 kg of carrier; ^b^ Citric acid by-product from rice (CABR) containing 91.0% dry matter, 19.8% crude protein, 3.9% ether extract, 11.9% crude fiber, 46.6% nitrogen-free extract, 0.43% calcium, 0.07% phosphorus, 0.05% methionine, 4005.7 kcal/kg gross energy.

**Table 2:** Effect of dietary inclusion with citric acid by-product from rice (CABR) on BWG, FI, FCR, and SR in Thai KKU 1 broiler chickens.

Parameter	Treatments					SEM	*p*-Value	Linier	Quadratic
	Control	3% CABR	6% CABR	9% CABR	12% CABR				
Initial weight (g/b)	32.77	32.73	32.80	32.89	32.64	0.222	0.954	0.0000	0.0020
Starter (1–21 days)									
BW (g/b)	384	392	3712	377	371.33	11.15	0.647	0.0010	0.0030
BWG (g/b)	351	360	339	344	338.69	11.17	0.650	0.0000	0.0000
FI (g/b)	526	515	541	544	542	14.40	0.500	0.0000	0.0000
FCR	1.51	1.43	1.60	1.59	1.62	0.0608	0.214	0.0000	0.0025
SR (%)	100	100	96.9	100	100	0.807	0.0528	0.0050	0.0060
Grower (22–49 days)									
BW (g/b)	1461 ^ab^	1475 ^a^	1423 ^abc^	1313 ^c^	1343 ^bc^	55.28	0.0408	0.0020	0.0030
BWG (g/b)	10,781 ^a^	1083 ^a^	1051 ^ab^	936.32 ^c^	972.12 ^bc^	44.63	0.0172	0.0030	0.0040
FI (g/b)	2171	2158	2091	2070	2313	77.64	0.0568	0.0010	0.0020
FCR	2.02 ^b^	1.99 ^b^	1.99 ^b^	2.22 ^a^	2.35 ^a^	0.0784	0.0011	0.0020	0.0030
SR (%)	93.8	95.3	96.9	95.3	98.4	4.92	0.903	NS	NS
Finisher (50–56 days)									
BW (g/b)	1774 ^a^	1770 ^a^	1715 ^ab^	1638 ^b^	1604 ^b^	42.96	0.4152	0.0020	0.0030
BWG (g/b)	312	295	290	325	260	19.90	0.415	0.0010	0.0020
FI (g/b)	875	866	946	905	885	30.90	0.245	0.0030	0.0040
FCR	2.87	2.97	3.30	2.82	3.42	0.277	0.142	0.0020	0.0030
SR (%)	100	100	100	96.8	100	1.16	0.0531	NS	NS
Overall period (1–56 days)									
BWG (g/b)	1741 ^a^	1737 ^a^	1680 ^ab^	1604 ^b^	1570 ^b^	40.8	0.031	0.0030	0.0040
FI (g/b)	3616	3566	3597	3738	3753	85.29	0.432	0.0010	0.0020
FCR	2.08 ^c^	2.05 ^c^	2.15 ^bc^	2.33 ^ab^	2.39 ^a^	0.06	0.004	0.0020	0.0030
SR (%)	93.1	95.3	93.8	92.2	98.4	3.45	0.755	NS	NS
Feed cost (USD/bird)	0.50	0.49	0.49	0.48	0.47	-	-	-	-

Dietary treatments consisted of levels of citric acid waste product from rice (CABR) in the diet at 0, 3, 6, 9, and 12% DM; ^a^–^c^ Means in the same row without a common letter are different at *p* < 0.05; SEM, standard error of means; BWG, body weight gain; FI, feed intake; FCR, feed conversion ratio; SR, survival rate. NS, nonsignificant.

**Table 3:** Effect of dietary inclusion of citric acid by-product from rice (CABR) on carcass and internal organs in Thai KKU 1 broiler chickens.

Parameter	Treatments					SEM	*p*-Value		
	Control	3% CABR	6% CABR	9% CABR	12% CABR			Linear	Quadratic
Live weight (g)	1787	1694	1704	1595	1568	75.1	0.128	NS	NS
Dressing percentage (%)	68.3 ^a^	67.7 ^ab^	67.8 ^a^	67.5 ^a^	63.8 ^b^	1.17	0.0074	0.0010	0.0020
External organs									
Breast (%)	24.0	25.3	23.6	23.8	23.2	1.50	0.741	NS	NS
Thigh (%)	17.2	18.1	17.1	17.4	18.3	2.18	0.975	NS	NS
Drumstick (%)	15.6	15.5	15.8	16.0	15.7	0.665	0.822	NS	NS
Wing (%)	12.9	13.0	13.9	13.7	14.0	0.520	0.571	NS	NS
Internal organs									
Liver (%)	2.86	3.15	3.07	3.43	3.63	0.508	0.747	NS	NS
Heart (%)	0.71	0.64	0.66	0.74	0.76	0.074	0.760	NS	NS
Pancreas (%)	0.36	0.36	0.33	0.34	0.39	0.040	0.605	NS	NS
Gizzard (%)	3.13	3.25	3.06	3.40	3.64	0.266	0.205	NS	NS
Abdominal fat (%)	2.21	2.55	1.56	1.78	1.66	0.497	0.641	NS	NS

Dietary treatments consisted of levels of citric acid waste product from rice (CABR) in the diet at 0, 3, 6, 9, and 12% DM; ^a^,^b^ Means in the same row without a common letter are different at *p* < 0.05; SEM, standard error of means. NS, nonsignificant.

**Table 4:** Effect of dietary inclusion of CABR on breast and thigh meat quality.

Parameter	Treatments					SEM	*p*-Value		
	Control	3% CABR	6% CABR	9% CABR	12% CABR			Linear	Quadratic
Breast meat									
Color									
L*	61.3	60.5	62.3	61.6	62.0	0.720	0.358	NS	NS
a*	11.0	11.9	10.4	10.0	10.3	0.890	0.487	NS	NS
b*	13.1 ^ab^	15.2 ^a^	11.3 ^b^	14.2 ^a^	11.2 ^b^	1.02	0.019	NS	NS
Drip loss (%)	6.01	5.83	5.49	5.89	5.98	0.570	0.954	NS	NS
Cooking loss (%)	17.3	17.0	18.4	16.6	19.0	1.40	0.618	NS	NS
Shear force (kg/cm^2^)	4.14	4.02	4.05	3.69	3.93	0.340	0.881	NS	NS
Thigh meat									
Color									
L*	60.6	61.4	60.3	61.3	62.5	1.02	0.430	NS	NS
a*	11.5	11.4	12.0	11.4	11.3	0.530	0.904	NS	NS
b*	9.09	10.8	8.51	10.6	8.87	1.10	0.401	NS	NS
Drip loss (%)	6.80	6.74	6.64	6.84	6.90	1.06	0.997	NS	NS
Cooking loss (%)	13.9	13.6	13.4	13.0	12.2	1.44	0.823	NS	NS
Shear force (kg/cm^2^)	3.81	3.56	3.28	3.99	3.73	0.430	0.318	NS	NS

Dietary treatments consisted of levels of citric acid waste product from rice (CABR) in the diet at 0, 3, 6, 9, and 12% DM; ^a^,^b^ Means in the same row without a common letter are different at *p* < 0.05; SEM, standard error of means; a*, redness; b*, yellowness, L*, lightness. NS, nonsignificant.

**Table 5:** Effects of the utilization of CABR on the meat composition of Thai KKU 1 broiler chickens.

Parameter	Treatments					SEM	*p*-Value		
	Control	3% CABR	6% CABR	9% CABR	12% CABR			Linear	Quadratic
Breast meat									
Dry matter (%)	27.5	30.0	30.0	28.1	29.3	0.582	0.056	0.038	0.070
Gross energy (kcal/kg)	4405 ^b^	4745 ^a^	4398 ^b^	4522 ^ab^	4816 ^a^	113.62	0.040	0.019	NS
Ether extract (%)	1.83 ^b^	3.48 ^a^	2.71 ^ab^	2.55 ^ab^	1.74 ^b^	0.440	0.048	0.035	NS
Crude protein (%)	64.8	74.2	78.6	78.5	78.4	8.55	0.157	0.015	NS
Thigh meat									
Dry matter (%)	27.0	23.3	28.8	26.5	24.8	1.60	0.098	0.045	0.082
Gross energy (kcal/kg)	5106 ^b^	5148 ^b^	5288 ^a^	5152 ^b^	5328 ^a^	19.3	0.003	0.000	0.015
Ether extract (%)	12.6	12.1	12.0	13.9	12.5	1.00	0.435	NS	NS
Crude protein (%)	67.4	66.7	72.5	71.4	73.0	4.46	0.550	NS	NS

Dietary treatments consisted of levels of citric acid waste product from rice (CABR) in the diet at 0, 3, 6, 9, and 12% DM; ^a^,^b^ Means in the same row without a common letter are different at *p* < 0.05; SEM, standard error of means. NS, nonsignificant.

## Data Availability

The original contributions presented in the study are included in the article, further inquiries can be directed to the corresponding author.
